# Water bodies are potential hub for spatio-allotment of cell-free nucleic acid and pandemic: a pentadecadal (1969–2021) critical review on particulate cell-free DNA reservoirs in water nexus

**DOI:** 10.1186/s42269-022-00750-y

**Published:** 2022-03-07

**Authors:** Bright Esegbuyota Igere, Hope Onohuean, Uchechukwu U. Nwodo

**Affiliations:** 1 SAMRC Microbial Water Quality Monitoring Centre, University of Fort Hare, Alice, 5700 Eastern Cape South Africa; 2 Applied and Environmental Microbiology Research Group, Department of Biochemistry and Microbiology, University of Fort Hare, Alice, 5700 Eastern Cape South Africa

**Keywords:** Membrane vesicles, Exogenous DNA, Extra-chromosomal DNA, Water nexus, Nucleic acid reservoir, Cell-free/particulate nucleic acid infections (cf/PNAI)

## Abstract

**Background:**

In recent times, there had been report of diverse particulate nucleic acid-related infections and diseases which have been associated with endemic, sporadic, and pandemic reports spreading within water nexus. Some of such disease cases were seldom reported in earlier years of technological advancement and research based knowledge-scape. Although the usefulness of water, wastewater treatment systems, water regulatory organizations and water re-use policy in compliant regions remains sacrosanct, it has been implicated in diverse gene distribution.

**Main body:**

A cosmopolitan bibliometric and critical assessment of cell-free DNA reservoir in water bodies was determined. This is done by analysing retrieved pentadecadal scientific publications in Scopus and Pubmed centre database, determining the twelve-monthly publication rates of related articles, and a content-review assessment of cell-free nucleic acids (cfNAs) in water environment. Our results revealed thirty-eight metric documents with sources as journals and books that conform to the inclusion criteria. The average reports/publication rate per year shows 16.7, while several single and collaborating authors are included with a collaboration index of 4.31. A zero average citation per document and citation per year indicate poor research interest and awareness.

**Short conclusion:**

It is important to note that a redirected interest to studies on cfNAs in water environments would encourage advancement of water treatment strategies to include specific approaches on the removal of cfNAs, membrane vesicles or DNA reservoirs, plasmids or extra-chromosomal DNA and other exogenous nucleic acids from water bodies. It may also lead to a generational development/improvement of water treatment strategies for the removals of cfNAs and its members from water bodies.

## Background

The water nexus as a relevant source of livelihood has been implicated as a hub for the distribution of diverse nucleic acid or genes especially as water treatment system and water reuse policy is implemented globally (Suzuki and Hoa 2012; Igere et al. 2021). Other related studies have also revealed the presence of diverse treatment chemical agents, bacteria, virus, protozoan, fungus and plankton of all kinds in wastewater release or effluents (Igere et al. 2021; Aminov 2011). Such treated/filtered and untreated water specimens have also revealed presence of particulate cell-free nucleic acids (PCFNAs) including plasmids or extra-chromosomal DNA, exogenous DNA (exDNA), phage and membrane vesicles (Abe et al. 2020; Woegerbauer et al. 2020). With the increasing reports of pandemics associated with PNAs from diverse surveillance data, the call to source track the origin of such PNA-borne infectious agents becomes eminent. PcfNAs are seen to have risen from incomplete chemical (chemical agents and antibiotics) breakdown of cellular components (organism) and worn-out tissues which gain their presence into water nexus via waste release (Woegerbauer et al. 2020; Toyofuku et al. 2019, 2017). These cell-free nucleic acid (cfNA)-based components have been linked with various health depleting concerns including resistance to antibiotics, evolving strains and virulence determinants which are probably shared via horizontal gene transfer (HGT) (Abe et al. 2020; Aminov 2011). Over the years, one notable area, where such cfNA has been reported in the literatures with relevant implications, is antibiotic resistant genes (ARGs) (Igere et al. 2020; Hashiguchi et al. 2019). These non-cellular nucleic acid materials which float in natural water nexus (particle) are become increasingly implicated in PNA related diseases which are expanding across multiple countries with varying severity amongst population of bacteria, viruses, protozoan, etc. (Woegerbauer et al. 2020; Hong et al. 2018). Such cfNA components, although in particulate state, are been acquired by most environmental organism which instil in them the capacity to survive harsh conditions of their respective environments. Investigators of nucleic acid in water bodies have frequently reported exchange of genes via HGT using microbial genetic cassette including mobile genetic elements, biofilms, vesicles mediated DNA, phage, transposon, cell-free/released DNA and other mobile integrative and conjugative elements (Abe et al. 2020; Woegerbauer et al. 2020; Calero-Caceres et al. 2019; Partridge et al. 2018; Carattoli 2013; Wozniak and Waldor 2010). It is important to note that such genetic components reside in regions that are not necessarily involved in genetic mobility (Partridge et al. 2018), but to elicit health-related concerns (Woegerbauer et al. 2020; Norton et al. 2013). As a matter of verity, there is a non-negligible quantity of nucleic acids carried as cell-free deoxyribonucleic acids (cfDNAs) in the form of extra-chromosomal DNA or plasmids, exDNA, phage, and membrane vesicle DNA which are probably been involve in disease situation or emergence of disease cases (Woegerbauer et al. 2020). The question of mechanism of sharing of exogenous nucleic acids in water bodies is another aspect of interest, which has arouse questions as water reuse policy and the fate of cfNAs impact other lives in both water bodies and environment. Although much attention has not been given to potential alternative mechanism of exogenous nucleic acid acquisition in water bodies, it has encouraged difficulties in estimating their true implications. In spite of these aforementioned cases of cfNAs in water bodies, studies on its dissemination has received poor research-based attention as well as its potential health associated risk or concern.

*Aims* It is to this end our study determines a critical assessment of cfNAs in water bodies as potential hub for emergence of PNA diseases and microbes with a view to appraising related studies and arousing interest on the removal of cfNAs from water bodies.

## Summary of Searched documents, methods and research design

### Reporting and protocol registration

This investigation applied the Preferred Reporting Items for Systematic Reviews and Meta-Analysis (PRISMA) protocols (Moher et al. 2009) which is submitted to the International Prospective Register of Systematic Reviews (PROSPERO). The study retrieved various reports of cell-free nucleic acids, membrane vesicles or MV, exogenous DNA and extra-chromosomal DNA in water bodies.

### Search strategy

In PubMed and Scopus databases, the search phrases (Wastewater AND Cell free nucleic acid OR extra-chromosomal DNA OR exogenous DNA OR membrane vesicles) were used to find datasets. Most often, various journals publish studies that were not extensively described and conducted. To eliminate such concerns, IBE and OH applied the Scopus and PubMed database after adequate consultations with UUN. The application of Scopus and PubMed database as source retrieval database was associated with verity of information, datasets with reputable study relevance and the public health relevance of studies in the specified database. All downloaded information was documents that conform with the condition for retrieval and inclusion such as research articles, editor letter, articles proceedings, and abstract review articles, whereas documents such as book chapters, book reviews news documents, opinions, and adverts were not included since they are not constituents of primary sources. In addition, the content search documents were also retrieved as PDF documents on articles titles and abstract focused on exDNA, cfNAs, membrane vesicles, extra-chromosomal DNA, wastewater effluent and water nexus. Authors collaboration, countries of study, countries collaboration and collaboration index of studies on cfNAs components in wastewater and water nexus were also accessed.

### Main text

#### Inclusion and exclusion criteria

Only articles that contain any of the search term or word(s); (Wastewater AND Cell free nucleic acid OR extra-chromosomal DNA OR exogenous DNA OR membrane vesicles) in the title/abstract were retrieved from the databases for a period of January 1969 to July 2021.

Duplicate documents were also removed, while author’s articles and other nonconforming documents to the applied inclusion criteria were not selected.

### Data analysis

Prior to data analysis, authors keywords, names, spelling errors and the appropriate Boolean were employed to extract relevant documents, and data were also normalised by IBE and OH. The datasets were saved in CSV format, combined in the excel file, and duplicate were removed. The clustered metric networks studied were built using a VOSviewer 1.6.13 optimized algorithm and the visualizing of similarity (VOS) protocol (Eck and Waltman 2007; Van Eck and Waltman 2007).

Whereas descriptive statistical methods were employed to examine the retrieved data, the results were presented in tables and charts as ranges, percentages, and distribution/frequencies.

### Investigators report and publication on cell-free nucleic acid in water nexus

Between the 50-year/jubileean period of studied articles (1969–2021), we collated annual scientific publication of diverse authors and journals which were grouped into 5 decadal period for the distribution of reports. It also reveals the frequency of related studies on cell-free nucleic acid (membrane vesicles, exogenous/extra-chromosomal nucleic acids) from diverse investigators, journals and interest-based personnel.

### Concerted actions of investigators on cell-free nucleic acid in water nexus

Reports on the various author’s interest and actions on Cell-free nucleic acid in water nexus were also retrieved to reveal the progress of studies and corrective steps to removing such non-cellular components from water bodies. It also shows the various countries that have embarked on related studies globally and the outcome of such studies. The study time span across 1969–2021, while unretrieved and nonconforming documents to the specified inclusion criteria were removed.

## Issues on results and discussion

**Fig. 1:**
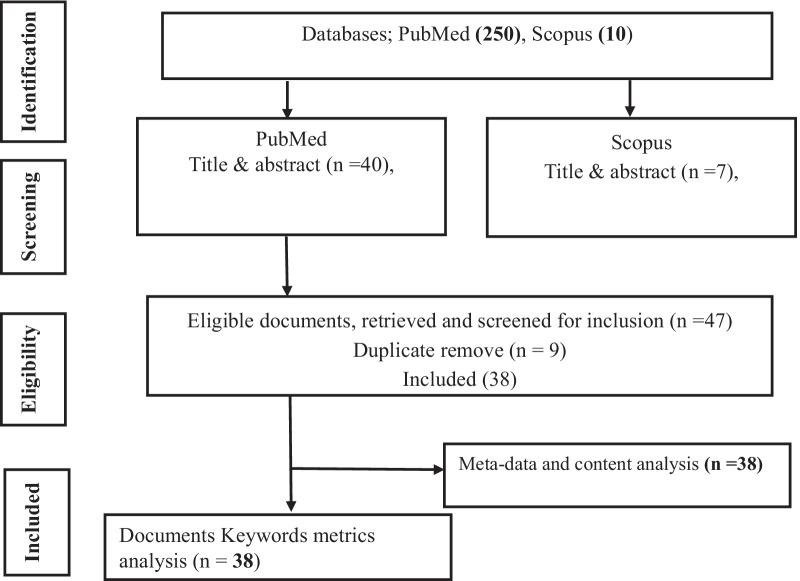
PRISMA strategy and flowchart of searching, reviewing and selecting of articles on Wastewater AND Cell free nucleic acid OR extra-chromosomal DNA OR exogenous DNA OR membrane vesicles

A total of two hundred and sixty documents were retrieved from Scopus and PubMed database as shown in Fig. 1 and Table 1 while the various datasets were analysed, respectively. Thirty-eight metric documents which conform to the inclusion criteria were both used for analysis and content review. These include: Sources (journals, books etc.) (34), average publication year and reports (16.7), included authors in related studies (157), single authors document (2), collaborating authors (155), authors index of collaboration: single (2), authors per document index (4.13), co-authors per documents index (4.42), collaboration index (4.31), whereas there are no average citation per document and average citation per year (0) while funding and supporting organization includes nih, US govt, non-phs, non-US govt, phs, etc. Figure 1 shows the PRISMA strategy and flow chat for searching, reviewing and selecting of articles. From the forgoing, it is clear that the rate of production of articles/books as well as the numbers of studies on cell-free nucleic acids, exogenous nucleic acids, membrane vesicles, etc., in wastewater release into the environment remains few. The fewness of reported publications on the subject was further affirmed by a null average citation per document and per year. This has also resulted very low single author’s index of collaboration, as well as multiple authors and countries collaboration index. The observed research supporting and funding organizations interest on related studies were also low affirming the low interest on studies of cell-free nucleic acids in water bodies.

**Table 1:** Main information about the dataset on Wastewater AND Cell free nucleic acid OR extra-chromosomal DNA

Description	Results
Time-span	1969:2020
Sources (journals, books, etc.)	34
DOCUMENTS	38
Average years from publication	16.7
Average citations per documents	0
Average citations per year per doc	0
References	1
DOCUMENT TYPES	
Comment; journal article	1
Comparative study; journal article; research support, non-us gov't	3
Journal article	12
Journal article; research support, nih, extramural	1
Journal article; research support, nih, extramural; research support, us gov't, non-phs	1
Journal article; research support, non-us gov't	10
Journal article; research support, non-us gov't; research support, us gov't, non-phs	3
Journal article; research support, non-us gov't; research support, us gov't, phs	2
Journal article; research support, non-us gov't; review	2
Journal article; research support, us gov't, non-phs; research support, us gov't, phs	1
Journal article; review	2
DOCUMENT CONTENTS	
Keywords plus (id)	291
Author's keywords (de)	291
AUTHORS	
Authors	157
Author appearances	168
Authors of single-authored documents	2
Authors of multi-authored documents	155
Authors collaboration	
Single-authored documents	2
Documents per author	0.242
Authors per document	4.13
Co-authors per documents	4.42
Collaboration index	4.31

phs, Public Health System; nih, National Institute of Health

### Investigators report and publication on cell-free nucleic acid in water nexus

**Fig. 2:**
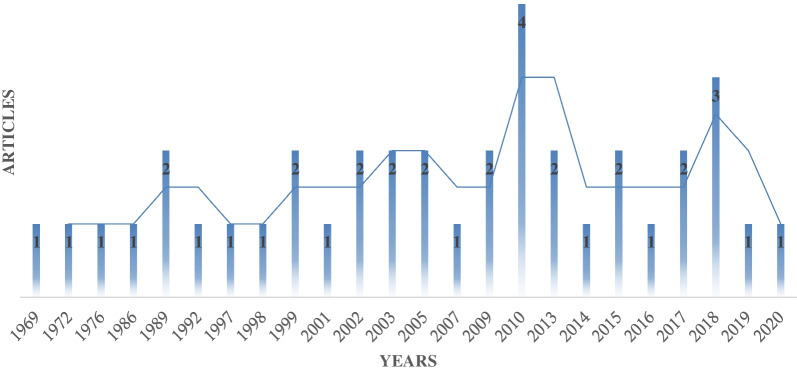
Overview of the year metrics on Wastewater AND Cell free nucleic acid OR extra-chromosomal DNA

The annual metrics of investigators and authors study on Cell-free nucleic acid (membrane vesicles, exogenous/extra-chromosomal nucleic acids) and water bodies globally is shown in Fig. 2. It can be observed from the above that a high proportion of such studies were conducted in 2010. The 5th decadal assessment and analysis of published articles showed that a high proportion of the documents were recorded/reported in the fourth decade (4), while the fifth decade ranked second in the publication frequency or reports (3). Also observed in the figure above is the silence in reports within some of the years under investigation, indicating that after the first report of cell-free nucleic acids in water bodies, interest on the study was not encouraged by research organizations; hence, research reports were not recorded in diverse countries and authors. This poor and/or low reporting state possess potential implications on the environments which are receiving the burden of cell-free nucleic acids release, since there are few recorded stride towards the removal of released cell-free nucleic acids.

The various authors and countries productivity or publication frequency on Cell-free nucleic acid (membrane vesicles, exogenous/extra-chromosomal nucleic acids) and water bodies globally are reported in Table 2. USA ranked the highest country with the highest frequency or numbers of articles published on the subject under review with a percentage publication rate of 34.21% (13), while France ranked the second with a percentage publication rate of 5.26%. Amongst the authors investigative studies on the subject, it was observed that Deutsch DR ranked the highest in the numbers of articles (13), while other authors queue under the decreasing ranking (Table 2). The sources of information were journals of life sciences and Biotechnology including ACS Synthetic biology, Biochemical and Biophysical Research Communications, Acta Neuropathologica, Biotechnology and Bioengineering, Animal Biotechnology etc. This has shown that in addition to the low or few studies on cell-free nucleic acids, the release of such nucleic acid continues to burden environmental wellness with low removal tendency and research-based interest.

**Table 2:** Most productive (Authors, Country, Affiliations and Sources/Journal) on Wastewater AND Cell free nucleic acid OR extra-chromosomal DNA

Authors	Articles	Articles % of 38	Sources	Articles	Articles % of 38	Affiliations	Articles	Articles % of 38	Country	Articles	Articles % of 38
Deutsch DR	13	7.89	ACS Synthetic biology	2	5.26	The Rockefeller University	10	26.32	USA	13	34.21
Fischetti VA	3	0.63	Biochemical and Biophysical Research Communications	2	5.26	Iowa State University	7	18.42	France	2	5.26
Utter B	3	0.63	Plos one	2	5.26	AMES	4	10.53	Australia	1	2.63
Cao M	2	0.39	Proceedings of the National Academy of Sciences of the United States of America	2	5.26	Kochi University	4	10.53	Brazil	1	2.63
Gill RT	2	0.33	Acta Neuropathologica	1	2.63	Latvian Biomedical Research and Study Centre	4	10.53	India	1	2.63
Seetharam AS	2	0.39	Animal Biotechnology	1	2.63	Tehran University of Medical Sciences	4	10.53	Iran	1	2.63
Severin AJ	2	0.39	Biochemical Society Transactions	1	2.63	Jackson Foundation	3	7.89	Israel	1	2.63
Shao Z	2	0.39	Biology letters	1	2.63	Naval Medical Research Center-Frederick	3	7.89	Italy	1	2.63
Adams A	1	0.33	Biotechnology and Bioengineering	1	2.63	Chalmers University of Technology	2	5.26	Japan	1	2.63
Al-anouti F	1	0.33	Blood Cells Molecules & Diseases	1	2.63	Universidade Federal Do Rio Grande Do Norte	2	5.26	Latvia	1	2.63

**Fig. 3:**
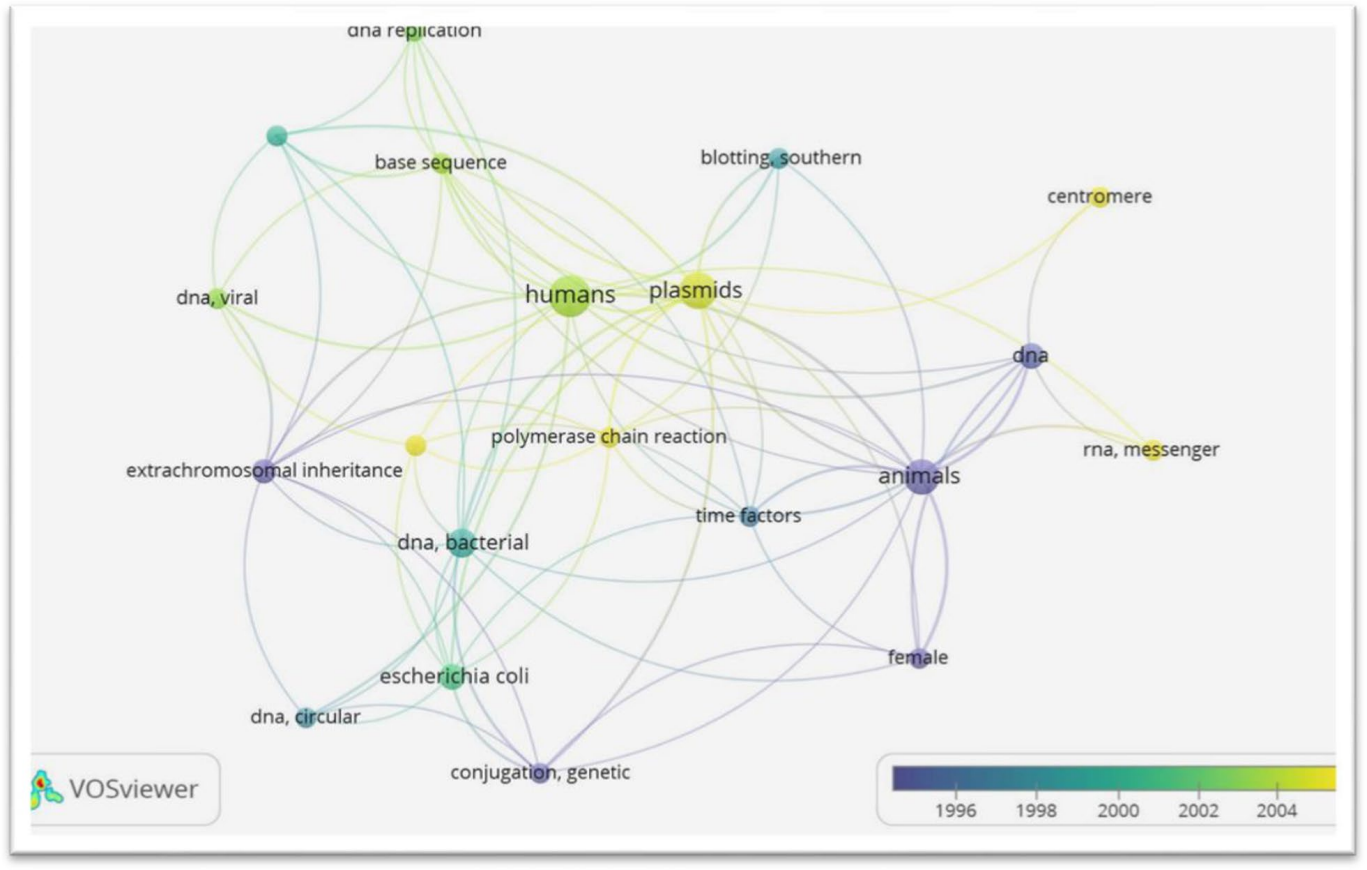
Analysis of all keywords co-occurrence on full counting by Vosviewer

From the analysis of keywords co-occurrence as shown in Fig. 3, 20 items met the threshold with a minimum number of occurrences of 3 for each, out of the 295 keywords. However, for each of the 20 keywords, the total strength of co-occurrence links with other keywords was calculated and the keywords with the greatest total linked strength were selected. These include plasmid, extra-chromosomal inheritance, DNA circular, DNA bacterial, based sequence, DNA replication, messenger RNA, etc. It can be deduced that between 1996 and 2004, there has been research focus on such particulate nucleic acid from DNA to messenger RNA although low. The potential trend of studies on released cell-free nucleic acid members today is directed at human, organisms, plasmid, messenger RNA, etc., where there had been potential implications. The network and link of these cell-free nucleic acid members may be associated with pandemics or epidemics if their removal from the environment is not encouraged. Suffice to say that some earlier studies have reported the presence of such cell-free nucleic acid members in water bodies (Woegerbauer et al. 2020). Such non-negligible quantities of cell-free nucleic acids possess potential health implications as well as disease emergence in any environment (Woegerbauer et al. 2020; Norton et al. 2013).

Table 3 shows the statistical distribution and occurrences of the keywords, their total linkage strength and the keywords co-occurrence analysis. It is observed that Human occurs the most relevant keyword (12) and a second position rank of linkage strength (22) indicating that human is the basic source to exogenous DNA or cell-free nucleic acids. The plasmid as a keyword ranked the second in occurrence (10), but it ranked the highest on the linkage strength (24) indicating that its linkage encourages transfer and/or sharing of exogenous nucleic acids. Suffice to say that plasmids are extra-chromosomal DNA which has both self-replicating tendency and antibiotic resistance genes. It also possesses the potential for horizontal sharing of its genetic constituents with other living organisms in water nexus and other environment (Ganesan et al. 2020; Ganesan and Vasudevan 2021; Duetsch et al. 2016). Other previous studies of some investigators have also revealed the acquisition of such relevant genetic characteristics via plasmids mediation by horizontal gene transfer (HGT). This is a potential source to the emergence of antibiotic resistance amongst microorganism as well as diverse pandemic particulate nucleic acids in the water nexus. With plasmid arising as the prevailing linkage strength, it is also supporting the previous studies as well as a potential indicator to the emerging nature of microbes within water bodies.

**Table 3:** Statistical distribution of occurrences, total link strength of all the keywords co-occurrence analysis

Keyword	Occurrences	Total link strength
Plasmids	10	24
Humans	12	22
Animals	9	21
DNA, bacterial	6	14
DNA	5	12
Extra-chromosomal inheritance	4	12
Base sequence	3	11
Time factors	3	11
Conjugation, genetic	3	10
Escherichia coli	5	10
Female	3	9
Molecular sequence data	3	9
Polymerase chain reaction	3	8
Dna, viral	3	7
Blotting, southern	3	6
Chromosomes, bacterial	3	6
DNA replication	3	6
DNA, circular	3	6
RNA, messenger	3	4
Centromere	3	2

Table 4 shows the general overview of studies on cell-free nucleic acid, authors, publishing journals, year of publication and PubMed citation ID. It may be adjudged that the few/low studies on cell-free nucleic acids in the environment and water bodies are attributable to poor interest or low awareness of the subject.

**Table 4:** An overview of studies on cell-free nucleic acid, authors, publishing journals, year of publication and PubMed citation ID

PMID	Title	Authors	Citation	First author	Journal/book	Publication year	PMCID	NIHMS ID	DOI
30013526	Extra-chromosomal DNA sequencing reveals episomal prophages capable of impacting virulence factor expression in Staphylococcus aureus	Deutsch DR, Utter B, Verratti KJ, Sichtig H, Tallon LJ, Fischetti VA	Front Microbiol. 2018 Jul 2;9:1406. https://doi.org/10.3389/fmicb.2018.01406. eCollection 2018	Deutsch DR	Front Microbiol	2018	PMC6036120		https://doi.org/10.3389/fmicb.2018.01406
30849712	The role of transposable elements and DNA damage repair mechanisms in gene duplications and gene fusions in plant genomes	Krasileva KV	Curr Opin Plant Biol. 2019 Apr;48:18–25. https://doi.org/10.1016/j.pbi.2019.01.004. Epub 2019 Mar 5	Krasileva KV	Curr Opin Plant Biol	2019			https://doi.org/10.1016/j.pbi.2019.01.004
31889879	Isolation and characterization of two novel plasmids pCYM01 and pCYM02 of Cylindrospermum stagnale	Ganesan V, Raja R, Hemaiswarya S, Carvalho IS, Anand N	Saudi J Biol Sci. 2020 Jan;27(1):535–542. https://doi.org/10.1016/j.sjbs.2019.11.017. Epub 2019 Nov 23	Ganesan V	Saudi J Biol Sci	2020	PMC6933252		https://doi.org/10.1016/j.sjbs.2019.11.017
27581613	Uncovering novel mobile genetic elements and their dynamics through an extra-chromosomal sequencing approach	Deutsch DR, Utter B, Fischetti VA	Mob Genet Elements. 2016 May 17;6(4):e1189987. https://doi.org/10.1080/2159256X.2016.1189987. eCollection 2016 Jul-Aug	Deutsch DR	Mob Genet Elements	2016	PMC4993567		https://doi.org/10.1080/2159256X.2016.1189987
28936238	Biodistribution, Uptake and Effects Caused by Cancer-Derived Extracellular Vesicles	Sadovska L, Santos CB, Kalniņa Z, Linē A	J Circ Biomark. 2015 Mar 25;4:2. https://doi.org/10.5772/60522. eCollection 2015 Jan-Dec	Sadovska L	J Circ Biomark	2015	PMC5572990		https://doi.org/10.5772/60522
29593241	Identification and DNA annotation of a plasmid isolated from Chromobacterium violaceum	Lima DC, Nyberg LK, Westerlund F, Batistuzzo de Medeiros SR	Sci Rep. 2018 Mar 28;8(1):5327. https://doi.org/10.1038/s41598-018-23708-5	Lima DC	Sci Rep	2018	PMC5871888		https://doi.org/10.1038/s41598-018-23708-5
20586577	Plasmid segregation: how to survive as an extra piece of DNA	Salje J	Crit Rev Biochem Mol Biol. 2010 Aug;45(4):296–317. https://doi.org/10.3109/10409238.2010.494657	Salje J	Crit Rev Biochem Mol Biol	2010			https://doi.org/10.3109/10409238.2010.494657
10208801	Development and evaluation of an extra chromosomal DNA-based PCR test for diagnosing bovine babesiosis	Salem GH, Liu X, Johnsrude JD, Dame JB, Roman Reddy G	Mol Cell Probes. 1999 Apr;13(2):107–13. https://doi.org/10.1006/mcpr.1998.0223	Salem GH	Mol Cell Probes	1999			https://doi.org/10.1006/mcpr.1998.0223
25843804	Extra-chromosomal DNA maintenance in Bacillus subtilis, dependence on flagellation factor FliF and moonlighting mediator EdmS	Hakumai Y, Shimomoto K, Ashiuchi M	Biochem Biophys Res Commun. 2015 May 15;460(4):1059–62. https://doi.org/10.1016/j.bbrc.2015.03.152. Epub 2015 Apr 3	Hakumai Y	Biochem Biophys Res Commun	2015			https://doi.org/10.1016/j.bbrc.2015.03.152
12212946	The food safety perspective of antibiotic resistance	McDermott PF, Zhao S, Wagner DD, Simjee S, Walker RD, White DG	Anim Biotechnol. 2002 May;13(1):71–84. https://doi.org/10.1081/ABIO-120005771	McDermott PF	Anim Biotechnol	2002			https://doi.org/10.1081/ABIO-120005771
28837318	Rapid Isolation of Centromeres from Scheffersomyces stipitis	Cao M, Seetharam AS, Severin AJ, Shao Z	ACS Synth Biol. 2017 Nov 17;6(11):2028–2034. https://doi.org/10.1021/acssynbio.7b00166. Epub 2017 Sep 5	Cao M	ACS Synth Biol	2017			https://doi.org/10.1021/acssynbio.7b00166
28391682	Centromeric DNA facilitates nonconventional yeast genetic engineering	Cao M, Gao M, Lopez-Garcia CL, Wu Y, Seetharam AS, Severin AJ, Shao Z	ACS Synth Biol. 2017 Aug 18;6(8):1545–1553. https://doi.org/10.1021/acssynbio.7b00046. Epub 2017 Apr 25	Cao M	ACS Synth Biol	2017			https://doi.org/10.1021/acssynbio.7b00046
24963913	Beyond the chromosome: the prevalence of unique extra-chromosomal bacteriophages with integrated virulence genes in pathogenic Staphylococcus aureus	Utter B, Deutsch DR, Schuch R, Winer BY, Verratti K, Bishop-Lilly K, Sozhamannan S, Fischetti VA	PLoS One. 2014 Jun 25;9(6):e100502. https://doi.org/10.1371/journal.pone.0100502. eCollection 2014	Utter B	PLoS One	2014	PMC4070920		10.1371/journal.pone.0100502
29602465	The presence of tumour extra-chromosomal circular DNA (ecDNA) as a component of liquid biopsy in blood	Khatami F, Larijani B, Tavangar SM	Med Hypotheses. 2018 May;114:5–7. https://doi.org/10.1016/j.mehy.2018.02.018. Epub 2018 Feb 26	Khatami F	Med Hypotheses	2018			https://doi.org/10.1016/j.mehy.2018.02.018
10382074	The degradation profile of extra-chromosomal circular DNA during cisplatin-induced apoptosis is consistent with preferential cleavage at matrix attachment regions	Schoenlein PV, Barrett JT, Welter D	Chromosoma. 1999 May;108(2):121–31. https://doi.org/10.1007/s004120050359	Schoenlein PV	Chromosoma	1999			https://doi.org/10.1007/s004120050359
19424419	Repair-mediated duplication by capture of proximal chromosomal DNA has shaped vertebrate genome evolution	Pace JK 2nd, Sen SK, Batzer MA, Feschotte C	PLoS Genet. 2009 May;5(5):e1000469. https://doi.org/10.1371/journal.pgen.1000469. Epub 2009 May 8	Pace JK 2nd	PLoS Genet	2009	PMC2671141		https://doi.org/10.1371/journal.pgen.1000469
1335022	Extra-chromosomal human immunodeficiency virus type 1 DNA forms in fresh peripheral blood lymphocytes and in two interleukin-2-independent T cell lines derived from peripheral blood lymphocytes of an asymptomatic seropositive subject	Titti F, Borsetti A, Federico M, Testa U, Meccia E, Samoggia P, Peschle C, Verani P, Rossi GB	J Gen Virol. 1992 Dec;73 ( Pt 12):3087–97. https://doi.org/10.1099/0022-1317-73-12-3087	Titti F	J Gen Virol	1992			https://doi.org/10.1099/0022-1317-73-12-3087
21170331	Relationships linking amplification level to gene over-expression in gliomas	Vogt N, Gibaud A, Almeida A, Ourliac-Garnier I, Debatisse M, Malfoy B	PLoS One. 2010 Dec 8;5(12):e14249. https://doi.org/10.1371/journal.pone.0014249	Vogt N	PLoS One	2010	PMC2999539		https://doi.org/10.1371/journal.pone.0014249
5009519	The timing of meiosis and DNA synthesis during early oogenesis in the toad, Xenopus laevis	Coggins LW, Gall JG	J Cell Biol. 1972 Mar;52(3):569–76. https://doi.org/10.1083/jcb.52.3.569	Coggins LW	J Cell Biol	1972	PMC2108647		https://doi.org/10.1083/jcb.52.3.569
20365401	Plasmid copy number noise in monoclonal populations of bacteria	Wong Ng J, Chatenay D, Robert J, Poirier MG	Phys Rev E Stat Nonlin Soft Matter Phys. 2010 Jan;81(1 Pt 1):011909. https://doi.org/10.1103/PhysRevE.81.011909. Epub 2010 Jan 14	Wong Ng J	Phys Rev E Stat Nonlin Soft Matter Phys	2010			https://doi.org/10.1103/PhysRevE.81.011909
23568537	Inverse metabolic engineering to improve Escherichia coli as an N-glycosylation host	Pandhal J, Woodruff LB, Jaffe S, Desai P, Ow SY, Noirel J, Gill RT, Wright PC	Biotechnol Bioeng. 2013 Sep;110(9):2482–93. https://doi.org/10.1002/bit.24920. Epub 2013 May 17	Pandhal J	Biotechnol Bioeng	2013			https://doi.org/10.1002/bit.24920
2927424	Effect of DNA damage on stable transformation of mammalian cells with integrative and episomal plasmids	Vos JM, Hanawalt PC	Mutat Res. 1989 Mar-May;220(2–3):205–20. https://doi.org/10.1016/0165-1110(89)90025-0	Vos JM	Mutat Res	1989			https://doi.org/10.1016/0165-1110(89)90025-0
20039170	Co-existence of multidrug-resistant and multidrug-susceptible strains of Pseudomonas aeruginosa from a single clinical isolate	Mahida K, Kwon DH	Curr Microbiol. 2010 Jul;61(1):19–24. https://doi.org/10.1007/s00284-009-9570-0. Epub 2009 Dec 29	Mahida K	Curr Microbiol	2010			https://doi.org/10.1007/s00284-009-9570-0
11515790	Glioblastoma-related gene mutations and over-expression of functional epidermal growth factor receptors in SKMG-3 glioma cells	Thomas C, Ely G, James CD, Jenkins R, Kastan M, Jedlicka A, Burger P, Wharen R	Acta Neuropathol. 2001 Jun;101(6):605–15. https://doi.org/10.1007/s004010000332	Thomas C	Acta Neuropathol	2001			https://doi.org/10.1007/s004010000332
19763421	Cloning and molecular characterization of a novel rolling-circle replicating plasmid, pK1S-1, from Bacillus thuringiensis subsp. kurstaki K1	Li MS, Roh JY, Tao X, Yu ZN, Liu ZD, Liu Q, Xu HG, Shim HJ, Kim YS, Wang Y, Choi JY, Je YH	J Microbiol. 2009 Aug;47(4):466–72. https://doi.org/10.1007/s12275-009-0020-2. Epub 2009 Sep 9	Li MS	J Microbiol	2009			https://doi.org/10.1007/s12275-009-0020-2
9844068	A novel method of extracting plasmid DNA from Helicobacter species	De Ungria MC, Tillett D, Neilan BA, Cox PT, Lee A	Helicobacter. 1998 Dec;3(4):269–77. https://doi.org/10.1111/j.1523-5378.1997.06085.pp.x-i1	De Ungria MC	Helicobacter	1998			https://doi.org/10.1111/j.1523-5378.1997.06085.pp.x-i1
23514143	Topological similarity between the 2 μm plasmid partitioning locus and the budding yeast centromere: evidence for a common evolutionary origin?	Jayaram M, Chang KM, Ma CH, Huang CC, Liu YT, Sau S	Biochem Soc Trans. 2013 Apr;41(2):501–7. https://doi.org/10.1042/BST20120224	Jayaram M	Biochem Soc Trans	2013			https://doi.org/10.1042/BST20120224
16093317	High-frequency gene targeting in Arabidopsis plants expressing the yeast RAD54 gene	Shaked H, Melamed-Bessudo C, Levy AA	Proc Natl Acad Sci U S A. 2005 Aug 23;102(34):12,265–9. https://doi.org/10.1073/pnas.0502601102. Epub 2005 Aug 10	Shaked H	Proc Natl Acad Sci U S A	2005	PMC1189313		https://doi.org/10.1073/pnas.0502601102
11997466	Genome-wide screening for trait conferring genes using DNA microarrays	Gill RT, Wildt S, Yang YT, Ziesman S, Stephanopoulos G	Proc Natl Acad Sci U S A. 2002 May 14;99(10):7033–8. https://doi.org/10.1073/pnas.102154799. Epub 2002 May 7	Gill RT	Proc Natl Acad Sci U S A	2002	PMC124523		https://doi.org/10.1073/pnas.102154799
17109398	Targeted gene delivery to differentiated skeletal muscle: a tool to study dedifferentiation	Morrison JI, Lööf S, He P, Aleström P, Collas P, Simon A	Dev Dyn. 2007 Feb;236(2):481–8. https://doi.org/10.1002/dvdy.21019	Morrison JI	Dev Dyn	2007			https://doi.org/10.1002/dvdy.21019
17148179	The evolution of a conjugative plasmid and its ability to increase bacterial fitness	Dionisio F, Conceição IC, Marques AC, Fernandes L, Gordo I	Biol Lett. 2005 Jun 22;1(2):250–2. https://doi.org/10.1098/rsbl.2004.0275	Dionisio F	Biol Lett	2005	PMC1626229		https://doi.org/10.1098/rsbl.2004.0275
816990	Extra-chromosomal DNA in chloramphenicol resistant myxococcus strains	Brown NL, Parish JH	J Gen Microbiol. 1976 Mar;93(1):63–8. https://doi.org/10.1099/00221287-93-1-63	Brown NL	J Gen Microbiol	1976			10.1099/00221287–93-1–63
12386375	Development of a Nuclear Export Signal Trapping Method for Isolating Genes with HIV Rev Activity	Zhang MJ, Dayton AI	J Biomed Sci. 1997 Nov-Dec;4(6):289–294. https://doi.org/10.1007/BF02258352	Zhang MJ	J Biomed Sci	1997			https://doi.org/10.1007/BF02258352
3796319	Plasmid-like properties of the four virulence-associated factors of Yersinia pestis	Tsukano H, Wake A, Sakakibara Y	Microbiol Immunol. 1986;30(9):837–48. https://doi.org/10.1111/j.1348-0421.1986.tb03011.x	Tsukano H	Microbiol Immunol	1986			https://doi.org/10.1111/j.1348-0421.1986.tb03011.x
12850476	Localization of HTLV-I tax proviral DNA in mononuclear cells	Zucker-Franklin D, Pancake BA, Najfeld V	Blood Cells Mol Dis. 2003 Jul-Aug;31(1):1–6. https://doi.org/10.1016/s1079-9796(03)00124-4	Zucker-Franklin D	Blood Cells Mol Dis	2003			https://doi.org/10.1016/s1079-9796(03)00124-4
2550376	Replication of latent Epstein-Barr virus genomes in normal and malignant lymphoid cells	Adams A, Pozos TC, Purvey HV	Int J Cancer. 1989 Sep 15;44(3):560–4. https://doi.org/10.1002/ijc.2910440331	Adams A	Int J Cancer	1989			https://doi.org/10.1002/ijc.2910440331
12604348	Double-stranded RNA can mediate the suppression of uracil phosphoribosyltransferase expression in Toxoplasma gondii	Al-Anouti F, Quach T, Ananvoranich S	Biochem Biophys Res Commun. 2003 Mar 7;302(2):316–23. https://doi.org/10.1016/s0006-291x(03)00172-4	Al-Anouti F	Biochem Biophys Res Commun	2003			https://doi.org/10.1016/s0006-291x(03)00172-4
5804897	Extra-chromosomal DNA in early stages of oogenesis in Acheta domesticus	Cave MD, Allen ER	J Cell Sci. 1969 May;4(3):593–609	Cave MD	J Cell Sci	1969			

It is important to note that the various activities of man including the various strategies on the control of diverse pathogenic organisms from systemic and superficial infections using biocidal agents encourage the release of cell-free nucleic acids/exogenous nucleic acids into the water bodies. The low research interest, low publications and/or poor awareness on the continuous release of cell-free nucleic acids from man’s activities into the environment is a potential time bomb. It is an explosive-based hotspot which may result disease as well as outbreak if appropriate research-based attention is not initiated.

## Conclusions

The knowledge on cell-free nucleic acids, its diverse members and knowledge-based interest appears to be dearth amongst researchers and investigators. This is revealed in the few reports and publications observed in the forgoing assessment. From the first report in man in 1948 by Mandel and Metais, to its first report in the environment in1969 by Cave and Allen, and in extension till 2021, there had been very few reports, yet there is a non-negligible release of such cell-free nucleic acids into the water milieu. This study has further revealed the poor interest and/or unawareness of such noxious components which are present in the water bodies and may be linked with emerging diverse disease cases. It is important to note that a redirected interest in cfNAs in water environment would encourage advancement of water treatment strategies to include specific approach to the removal of cell-free nucleic acids, membrane vesicles or DNA reservoirs, plasmids or extra-chromosomal DNA and other exogenous nucleic acids from water bodies. The interest on removal of cfNAs would also reduce the potential sharing of nucleic acids by diverse microbial strains in the environment which may also help to control/reduce occurrence of pandemic. In addition, it would also result in a reduction in HGT, metagenomic detection of exogenous nucleic acids and reduction in the mechanism of nucleic acids acquisition by cells and may lead to generational development of water treatment strategies.

## Data Availability

The datasets used for this study are available from the corresponding author on reasonable request.

## Abbreviations

PROSPERO: Prospective Register of Systematic Reviews
exDNA: Exogenous deoxyribonucleic acid
cfNAs: Cell-free nucleic acids
MV: Membrane vesicles
PcfNAs: Particulate cell-free nucleic acids
PRISMA: Preferred Reporting Items for Systematic Reviews and Meta-Analysis
PNAI: Particulate nucleic acid infections

## References

[CR1] Abe K, Nomura N, Suzuki S (2020). Biofilms: hot spots of horizontal gene transfer (HGT) in aquatic environments, with a focus on a new HGT mechanism. FEMS Microbiol Ecol.

[CR2] Aminov RI (2011). Horizontal gene exchange in environmental microbiota. Front Microbiol.

[CR3] Balcazar JL, Subirats J, Borrego CM (2015). The role of biofilms as environmental reservoirs of antibiotic resistance. Front Microbiol.

[CR4] Calero-Caceres W, Ye M, Balcazar JL (2019). Bacteriophages as environmental reservoirs of antibiotic resistance. Trends Microbiol.

[CR5] Carattoli A (2013). Plasmids and the spread of resistance. Int J Med Microbiol.

[CR6] Duetsch M, Pfahl S, Wernli H (2016) The influence of weather systems on interannual isotopic variability in a 10-year high resolution simulation of stable water isotopes over Europe. In: AGU fall meeting abstracts 2016 December, Vol 2016, pp PP23E-08

[CR7] Eck NJ, Waltman L. (2007a). VOS: a new method for visualizing similarities between objects. In: Advances in data analysis. Springer, Berlin, Heidelberg, pp 299–306

[CR8] Ganesan V, Raja R, Hemaiswarya S, Carvalho IS, Anand N (2020). Isolation and characterization of two novel plasmids pCYM01 and pCYM02 of Cylindrospermum stagnale. Saudi J Biol Sci.

[CR9] Ganesan S, Vasudevan N (2021). Genetically modified microbial biosensor for detection of pollutants in water samples. Environmental biotechnology 3.

[CR10] Hashiguchi TC, Ouakrim DA, Padget M, Cassini A, Cecchini M (2019). Resistance proportions for eight priority antibiotic-bacterium combinations in OECD, EU/EEA and G20 countries 2000 to 2030: a modelling study. Eurosurveillance.

[CR11] Hong PY, Julian TR, Pype ML, Jiang SC, Nelson KL, Graham D, Pruden A, Manaia CM (2018). Reusing treated wastewater: consideration of the safety aspects associated with antibiotic-resistant bacteria and antibiotic resistance genes. Water.

[CR12] Igere BE, Okoh AI, Nwodo UU (2020). Antibiotic Susceptibility testing reports: a basis for environmental/epidemiological surveillance and infection control amongst environmental *Vibrio cholerae*. Int J Environ Res Pub Health.

[CR13] Igere BE, Igolukumo BB, Eduamodu CE, Odjadjare EO (2021). Multi-drug resistant *Aeromonas* species in Annelida: An evidence of pathogen harbouring leech in recreation water nexus of Oghara Nigeria environs. Sci Afr.

[CR14] Moher D, Liberati A, Tetzlaff J, Altman DG (2009). Prisma Group. Reprint—preferred reporting items for systematic reviews and meta-analyses: the PRISMA statement. Phys Therapy.

[CR15] Norton SE, Lechner JM, Williams T, Fernando MR (2013). A stabilizing reagent prevents cell-free DNA contamination by cellular DNA in plasma during blood sample storage and shipping as determined by digital PCR. Clin Biochem.

[CR16] Partridge SR, Kwong SM, Firth N, Jensen SO (2018). Mobile genetic elements associated with antimicrobial resistance. Clin Microbiol Rev.

[CR17] Suzuki S, Hoa PTP (2012). Distribution of quinolones, sulfonamides, tetracyclines in aquatic environment and antibiotic resistance in Indochina. Front Microbiol.

[CR18] Suzuki S, Nakanishi S, Tamminen M, Yokokawa T, Sato-Takabe Y, Ohta K, Chou HY, Muziasari WI, Virta M (2019). Occurrence of sul and tet (M) genes in bacterial community in Japanese marine aquaculture environment throughout the year: profile comparison with Taiwanese and Finnish aquaculture waters. Sci Total Environ.

[CR19] Toyofuku M, Cárcamo-Oyarce G, Yamamoto T, Eisenstein F, Hsiao CC, Kurosawa M, Gademann K, Pilhofer M, Nomura N, Eberl L (2017). Prophage-triggered membrane vesicle formation through peptidoglycan damage in Bacillus subtilis. Nat Commun.

[CR20] Toyofuku M, Nomura N, Eberl L (2019). Types and origins of bacterial membrane vesicles. Nat Rev Microbiol.

[CR21] Van Eck NJ, Waltman L (2007). Bibliometric mapping of the computational intelligence field. Internat J Uncertain Fuzziness Knowl-Based Syst.

[CR22] Woegerbauer M, Bellanger X, Merlin C (2020). Cell-free DNA: an underestimated source of antibiotic resistance gene dissemination at the interface between human activities and downstream environments in the context of wastewater reuse. Front Microbiol.

[CR23] Wozniak RA, Waldor MK (2010). Integrative and conjugative elements mosaic mobile genetic elements enabling dynamic lateral gene flow. Nat Rev Microbiol.

